# Kicking in or kicking out? The role of the individual motor expertise in predicting the outcome of rugby actions

**DOI:** 10.3389/fpsyg.2023.1122236

**Published:** 2023-03-03

**Authors:** Simone Paolini, Maria Chiara Bazzini, Marco Rossini, Doriana De Marco, Arturo Nuara, Paolo Presti, Emilia Scalona, Pietro Avanzini, Maddalena Fabbri-Destro

**Affiliations:** ^1^Department of Surgery and Medicine, University of Parma, Parma, Italy; ^2^Institute of Neuroscience, National Research Council of Italy (CNR), Parma, Italy; ^3^Italian Rugby Federation (FIR), Rome, Italy; ^4^Department of Medical and Surgical Specialties, Radiological Sciences and Public Health (DSMC), University of Brescia, Brescia, Italy; ^5^Humanitas Clinical and Research Center, Milan, Italy

**Keywords:** action prediction, mirror mechanism, sports, Action Observation Training (AOT), motor repertoire

## Abstract

In sports, understanding others’ actions represents a fundamental skill that allows players to predict the outcome of teammates’ and opponents’ actions and counteract them properly. While it is well known that motor expertise sets better premises for predicting the result of an observed sports action, it remains untested whether this principle applies to a team where players cover different positions that imply different motor repertoires. To test this hypothesis, we selected rugby as a paradigmatic example in which only one or two players out of 22 train and perform placed kicks. We administered a placed kick outcome prediction task to three groups of participants, namely, rugby kickers, rugby non-kickers, and controls, thus spanning over different combinations of motor expertise and visual experience. Kickers outperformed both their non-kicking teammates and controls in overall prediction accuracy. We documented how the viewpoint of observation, the expertise of the observed kicker, and the position of the kick on the court influenced the prediction performance across the three groups. Finally, we revealed that within rugby players, the degree of motor expertise (but not the visual experience) causally affects accuracy, and such a result stands even after accounting for the level of visual experience. These findings extend the role of motor expertise in decoding and predicting others’ behaviors to sports teammates, among which every member is equipped with a position-specific motor repertoire, advocating for new motor training procedures combining the gestures to-be-performed with those to-be-faced.

## 1. Introduction

In sports, players constantly interact with other individuals, and the individual ability especially in team sports relies onto the capacity to understand and predict the teammates’ or opponent’s actions and outcomes, thus counteracting them properly (Brenton and Müller, 2018; Rizzolatti et al., 2021). The discovery of the mirror mechanism provided us with the neurophysiological mechanism underlying both these motor and cognitive functions, showing that action observation activates in the observer the same motor neural circuits recruited during the execution of that action (Rizzolatti and Sinigaglia, 2010; Rizzolatti et al., 2014).

An essential condition making the mirror mechanism effective is that the observed action has to be represented within the observer’s motor repertoire. The proof of such property was provided by Buccino et al. (2004), who showed that the fronto-parietal networks of subjects are activated by the observation of actions belonging to their motor repertoire (e.g., biting) regardless of the agent doing the action (dog, monkey, or human). The same networks did not respond to the observation of activities out of the subject’s motor repertoire (e.g., barking or lip-smacking). Another piece of evidence was provided by Calvo-Merino et al. (2005), showing that the activation of the mirror mechanism relies on matching with the individual motor expertise. In other words, the observed action is mapped onto my motor system, according to my motor skills.

The case of sports actions does not except this scenario. Indeed, during the last decades, many studies have highlighted that both motor expertise and visual experience may lead to earlier and more accurate predictions of action outcomes (Aglioti et al., 2008; Urgesi et al., 2012; Gabbett and Abernethy, 2013; Makris and Urgesi, 2015; Loffing and Cañal-Bruland, 2017). Overall, in sports, two different approaches can be envisioned during action prediction: the predictions can be based on visual cues (e.g., the ball trajectory), typical of expert observers (e.g., coaches and sports journalists), or on visuomotor cues (e.g., body kinematics and postures of the opponents), characteristic of expert athletes (Abernethy, 1991; Abernethy et al., 2008; Aglioti et al., 2008; Urgesi et al., 2012; Runswick et al., 2020). This latter aspect has been documented using both temporally- or – spatially occluded paradigms, both indicating the superiority of experts vs. novices in outcome prediction (Causer et al., 2017).

In this picture, an open issue is how the different team members in team sports exploit these approaches. Indeed, the matter of visuomotor expertise has been mainly considered as a dichotomy between experienced players and novices (Abernethy et al., 2008; Aglioti et al., 2008; Gabbett and Abernethy, 2013; Runswick et al., 2020), lacking an investigation of whether the individual motor repertoire intrinsic to a specific position modulates the capacities of action prediction.

Rugby offers an ideal scenario to investigate this issue as the 15 players on the court cover different positions implying extremely diversified motor abilities. The goalkicker represents a paradigmatic case. Indeed, only one player covers this position in a 15-player team, and their performance significantly impacts the game’s outcome. In addition, being a closed skill (i.e., individual and uncontested action) placed kicks are easy to use in experimental designs as they involve only one player and can be observed from multiple viewpoints. Alongside, previous literature argued that action prediction functions are usually more involved in open-skill contexts in which opponents are present (Bianco et al., 2017; Gu et al., 2019; Costa et al., 2023) than in closed skills. However, it is worth noting that also closed-skill scenarios require some degree of predictive abilities, as specific outcomes (the ball hitting the goalposts in rugby or saved by the goalkeeper in soccer) could require players to promptly react. The extreme specificity of the kicking motor expertise within rugby teams and its nature of “closed skill” led us to select kicking actions as the best stimuli for testing the role of motor expertise in predicting kick outcomes among rugby players. While former studies investigated the kinematic features of placed kicks (Flemmer and Flemmer, 2015; Atack et al., 2019; Bezodis et al., 2019), none addressed whether expert kickers better recognize those features compared to their teammates.

Starting from these premises, our study aims to evaluate whether rugby kickers are better at predicting placed kick outcomes than naïve subjects and their teammates covering different positions. Such a comparison would allow us to disentangle the contribution that visual experience and motor expertise bring into the action outcome prediction, ultimately guiding the development of new training procedures in team sports and further proving how our daily interactions rely on the dialogue between reciprocal sets of motor competencies.

## 2. Materials and methods

### 2.1. Participants

An *a priori* power analysis with G-Power 3.1 was conducted to define the sample size suitable for a within-between-subjects ANOVA design, including three groups, namely Rugby Kickers (RK), Rugby Non-Kickers (RNK), and Controls (CTRL). The output showed a minimum sample size of 78 participants (26 for each group) with an α = 0.05, power β = 0.85, and an effect size *f* = 0.70 per recommendations of Cohen (1988). Considering possible drop-outs and participants’ technical issues, we increased the group numerosity by 50%, i.e., 39.

Participants were recruited *via* the Prolific platform^1^ and redirected to the Gorilla Experiment Builder platform^2^ to carry out the experiment. We indicated a preference for people playing or having played rugby, but we had no chance to control the specific position covered by participants on the court. Thus, a preliminary questionnaire was administered to participants about their motor expertise and visual experience with sports (e.g., *Have you ever played sports? Which ones?*; *Have you ever seen sports competitions? Which ones?*).

If participants did not select rugby as a practiced sport, they were included in the CTRL group. Conversely, participants choosing rugby were administered additional questions about their position in the team (e.g., *Have you ever played as a kicker?*) to further subdivide them into RK and RNK. Finally, we asked them to rank their motor expertise with placed kicks (e.g., *How many placed kicks have you attempted in your career?*) and their specific visual experience with rugby (see Table 1). The recruitment was stopped only once all three groups reached at least 39 participants.

**Table 1:** Descriptive characteristics of the motor expertise and visual experience of the total sample.

Motor expertise (Overall)										
Group (*N* = 164)	No sport	Basket	Soccer (5)	Soccer (11)	Padel	Handball	Volleyball	Rugby	Tennis	Others
RK (39)	-	59.0%	74.4%	64.1%	33.3%	28.2%	64.1%	100.0%	53.8%	53.8%
RNK (49)	-	63.3%	53.1%	59.2%	12.2%	34.7%	67.3%	100.0%	49.0%	34.7%
CTRL (76)	9.2%	35.5%	43.4%	36.8%	7.9%	11.8%	60.5%	-	31.6%	50.0%
Visual experience (Overall)										
	No sport	Basket	Soccer (5)	Soccer (11)	Padel	Handball	Volleyball	Rugby	Tennis	Others
RK (39)	-	69.2%	41.0%	89.7%	23.1%	33.3%	74.4%	94.9%	74.4%	-
RNK (49)	4.1%	67.3%	18.4%	89.8%	8.2%	18.4%	77.6%	83.7%	71.4%	6.1%
CTRL (76)	2.6%	40.8%	7.9%	77.6%	2.6%	2.6%	48.7%	31.6%	44.7%	10.5%
Motor expertise (Rugby)										
		0	*n* < 5	5 < *n* < 50	50 < *n* < 100	100 < *n* < 500	500 < *n* < 1,000	*n* > 1,000		
	RK (30)	-	-	33.3%	33.3%	33.3%	-	-		
	RNK (49)	59.2%	22.4%	18.4%	-	-	-	-		
	CTRL (76)	-	-	-	-	-	-	-		
Visual experience (Rugby)										
		0	*n* < 5	5 < *n* < 50	50 < *n* < 100	100 < *n* < 500	500 < *n* < 1,000	*n* > 1,000		
	RK (38)	-	-	10.5%	13.2%	42.1%	23.7%	10.5%		
	RNK (39)	-	-	23.1%	10.3%	25.6%	25.6%	15.4%		
	CTRL (76)	50.0%	26.3%	17.1%	1.3%	1.3%	1.3%	-		

In the table’s upper lines, participants’ motor expertise is reported, i.e., which sports (if one or more) they have practiced or still practice today. The subsequent lines show participants’ visual experience, i.e., which sports matches they have watched in their life in the stadium and, for example, on television. Participants could select one or more sports both in visual and motor questionnaires. The lower lines focused on the rugby-specific motor expertise, i.e., how many placed kicks participants have executed in their careers (nine kickers were removed because they had not provided information about their motor expertise). Finally, the rugby-specific visual experience is reported, i.e., how many rugby matches participants have watched in their life (one kicker and 10 non-kickers were removed because they had provided misleading data). RK, Rugby Kickers; RNK, Rugby Non-Kickers; and CTRL, control group.

The final sample consisted of 164 participants: RK (*n* = 39, mean age 28.2 ± 7.2), RNK (*n* = 49, mean age 32.9 ± 11.4), and CTRL (*n* = 76, mean age 28.8 ± 9.2). The study was approved by the institutional ethical committee [Commissione per l’Etica e l’Integrità nella Ricerca—National Research Council (CNR), n. 00111709/2022, 15.02.2022]. It was conducted according to the principles expressed in the Declaration of Helsinki. The players in the videos gave written authorization to use their images, and the participants provided written informed consent.

### 2.2. Stimuli and experimental design

**Figure 1:**
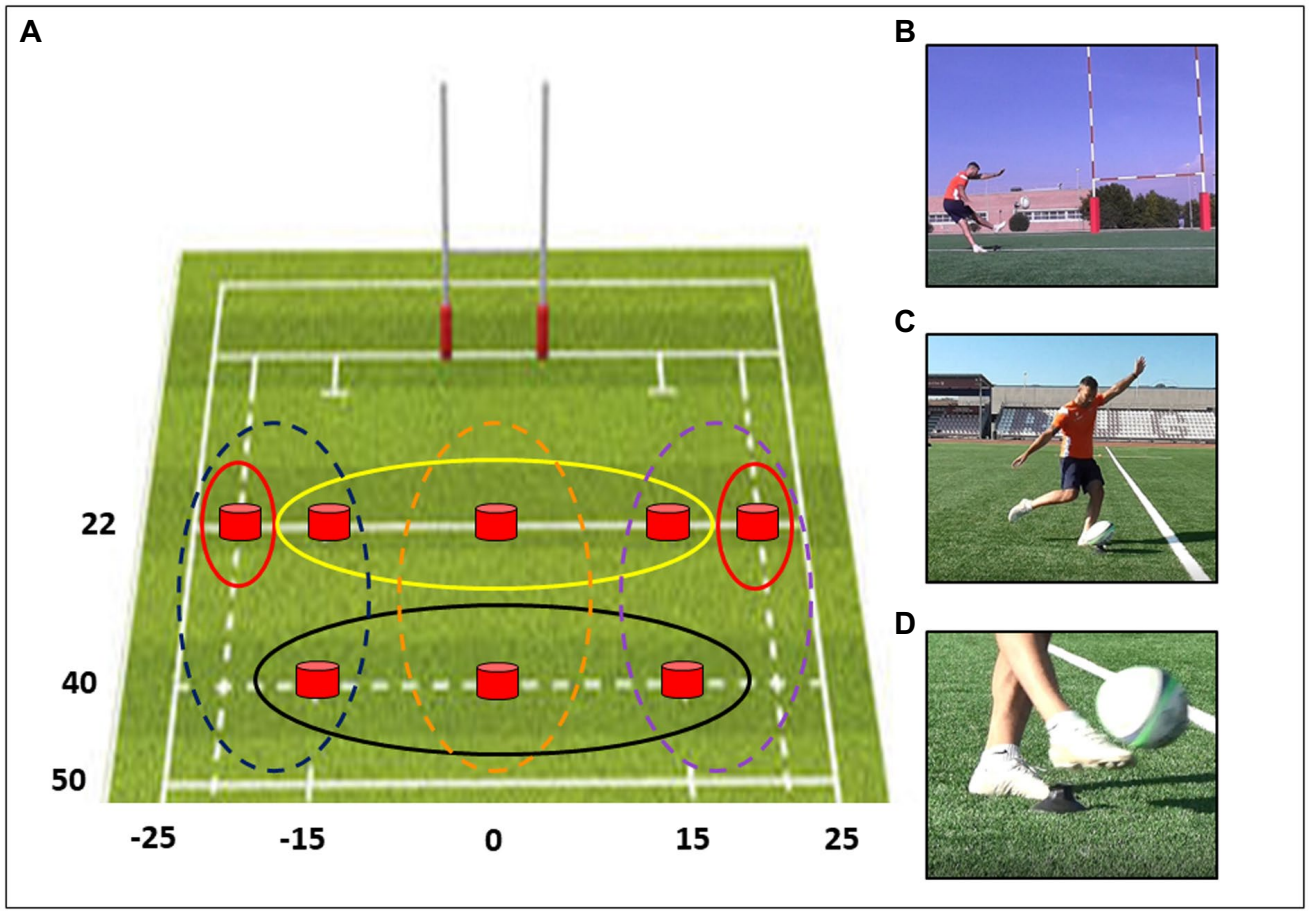
**(A)** shows a top view of the rugby mid-field, with the goalposts depicted on top. Numbers along the vertical and horizontal axes indicate in meters the distance of the kick positions from the goalposts line and the midline, respectively. Negative numbers refer to the left side of the court, and positive numbers to the right side, respectively. Red buttons indicate the eight positions from which placed kicks were executed. Red, yellow, and black circles refer to lateral, near, and distant zones. Blue, orange, and purple dotted circles refer to left-side, central and right-side positions on the court, respectively. **(B–D)** show the three perspectives from which a single placed kick was videotaped: back-view **(B)**, lateral **(C)**, and ball-only **(D)**.

To explore the capacity of predicting the outcome of observed placed kicks, we videotaped a training session of four kickers from an Italian Rugby team and later selected 144 videos of such gestures (lasting 2–7 s, stopped 200 ms after the foot-ball contact) according to three main factors (see Figure 1):

- Perspective: All kicks were simultaneously recorded from three different views, namely back-view, lateral, and ball-only, each including various degrees of visual information. The back-view displayed the full body of the kicker together with the goalposts. The lateral perspective framed only the kicker’s body without the goalposts, while the ball-only view exclusively showed the ball and its contact with the foot.
- Zone: Kicks were performed from eight positions on the rugby field (see red dots in Figure 1), further grouped in three different zones, namely near, lateral, and distant (depicted as red, yellow, and black circles in Figure 1) regardless of the laterality. The rationale was to test whether outcome prediction differs according to the field zones.
- Player: Four right-footed rugby players from an Italian professional Rugby team, two seniors (P1 and P2), and two juniors (under 18 years, namely P3 and P4), all serving as kickers in their respective teams, were videotaped, and their performance has been used as stimuli. This factor was intended to evaluate whether the observer’s outcome prediction varies along with the expertise of the observed model.

As we intended to design an outcome discrimination task where participants had to predict the kick outcome, i.e., whether the ball would have crossed the goalposts or not (namely kicking in/kicking out), we had to include both failed and successful kicks from any zone and by any players. We used two repetitions for any factorial combinations, so the overall design was composed of 144 videos, i.e., 3 perspectives × 3 zones × 4 players × 2 outcomes × 2 repetitions.

### 2.3. Data scoring

For each participant and trial, we first evaluated whether the response had been given before or after the contact between the foot and the ball. In the former case, no kick observation occurred. Thus, we excluded these trials as we had no chance to control the amount of information driving the performance in action prediction. In addition, participants with an exceedingly high rate of anticipated responses (>48, i.e., 33%) were excluded from the experimental sample.

On the final sample, we first computed the overall accuracy (i.e., percentage of correct answers) for each participant and the prevalence of kicking-in or kicking-out responses. As participants had a bias toward the kicking-in outcome (56.7 vs. 43.2%), we opted for computing the accuracy of each participant as *d* prime (*d*’). According to the Signal Detection Theory (SDT; Swets et al., 1961), *d*’ represents an indicator of the raters’ ability to recognize the true presence or absence of the characteristic being judged (Stanislaw and Todorov, 1999; Grant et al., 2017). It is calculated considering the hit rate and the false-alarm (FA) rate using the formula d’ = Z_Hit_−Z_FA_, where Z is the standardized score that allows for the comparison of measures with different ranges of absolute values (Macmillan and Creelman, 1990; Haatveit et al., 2010). A *d*’ = 0 means that participants reached 50% accuracy in both outcomes (kicking-in, kicking out), so they lay on a high randomness level. Positive *d*’ indicates that the participant predicted the kick outcome above chance.

### 2.4. Statistical analyses

All the analyses were conducted using *d*’ scores. A one-way ANOVA was initially undertaken to evaluate whether the overall accuracy differed among groups. *Post hoc* were Bonferroni corrected to account for multiple comparisons.

Subsequently, we applied three mixed ANOVA to investigate the main effect and possible interaction with Group of all our within-group factors (Perspective, Zone, and Player). A planned comparisons design (two-sample *t*-tests) was applied to highlight specific differences among groups (i.e., RK vs. RNK, RK vs. CTRL, and RNK vs. CTRL) in outcome prediction under different within-subjects levels. Bonferroni correction was further applied to the value of *p* to account for multiple comparisons, and Cohen’s *d* was calculated as a measure of effect size.

Finally, a multiple regression analysis was conducted exclusively on the rugby players to test whether the motor expertise and visual experience have a predictive role on the participants’ capacity to estimate placed kicks outcome. Of note, using a multiple regression model also allowed us to verify whether the association between motor expertise and outcome prediction stands after modeling the contribution due to the visual experience.

## 3. Results

The final sample was composed of 125 subjects (35 RK, 26 RNK, and 64 CTRL), as 30 had to be excluded because of the too high number of anticipated answers, and nine provided misleading information for the group assignment (e.g., never played rugby and never watched rugby matches but attempted more than 1,000 placed kicks).

**Figure 2:**
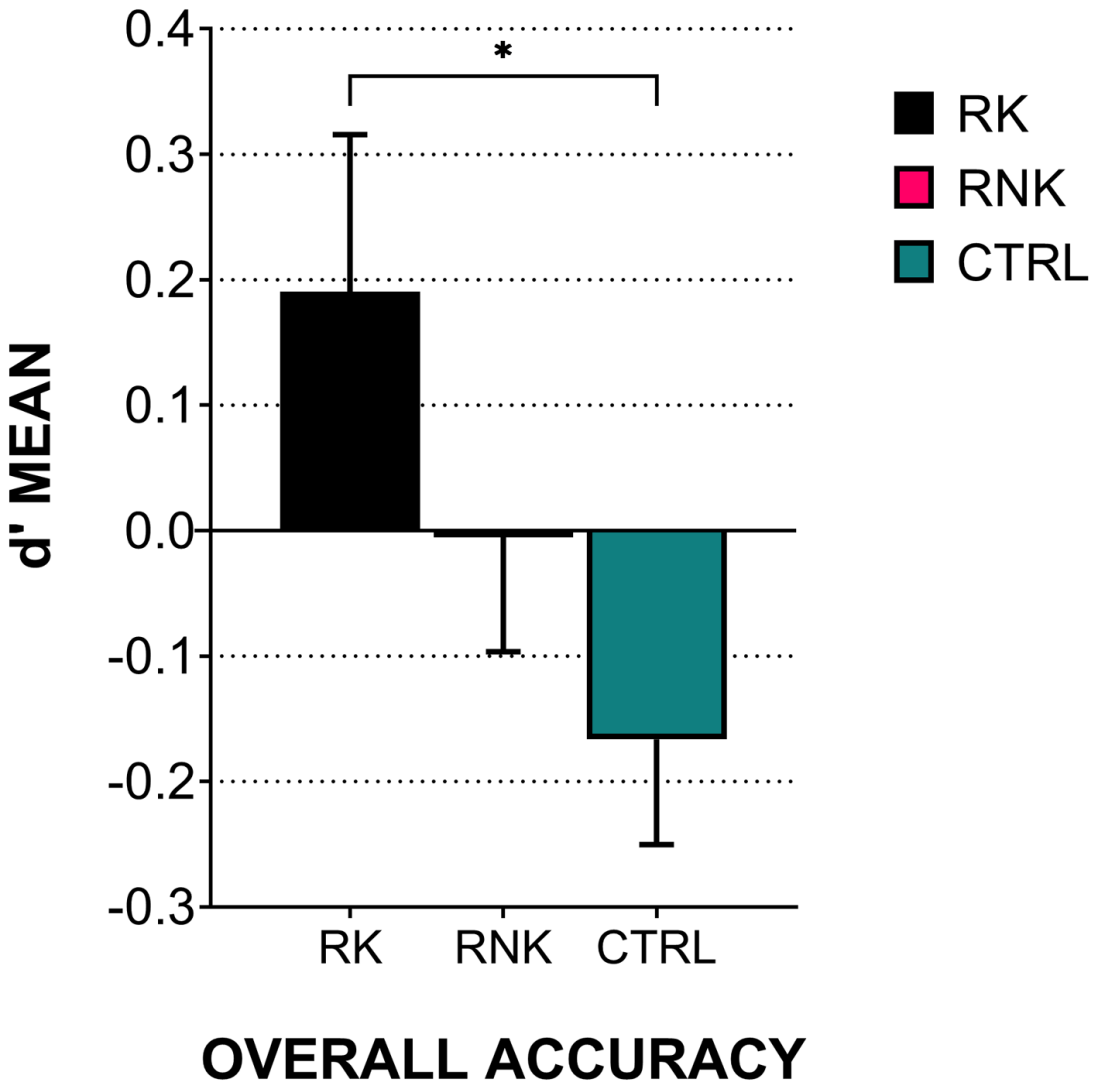
Overall accuracy (OA) among groups. The histograms indicate the average d’ scores, error bars indicate standard error. Asterisks indicate *post hoc* values of *p* < 0.05. RK, Rugby Kickers; RNK, Rugby Non-Kickers; and CTRL, control group.

The one-way ANOVA on the overall accuracy (OA) pointed out a main effect of the Group in kick outcome prediction [*F*_(2,122)_ = 3.38, *p* = 0.03]. As shown in Figure 2, RK significantly outperformed CTRL [*M* RK = 0.19, *M* CTRL = − 0.16; *t*_(97)_ = 2.43, *p*_(corrected)_ = 0.04, Cohen’s *d* = 0.51], while RNK positioned at an intermediate score (*M* RNK = − 0.005), with no differences in the comparisons with other groups.

**Figure 3:**
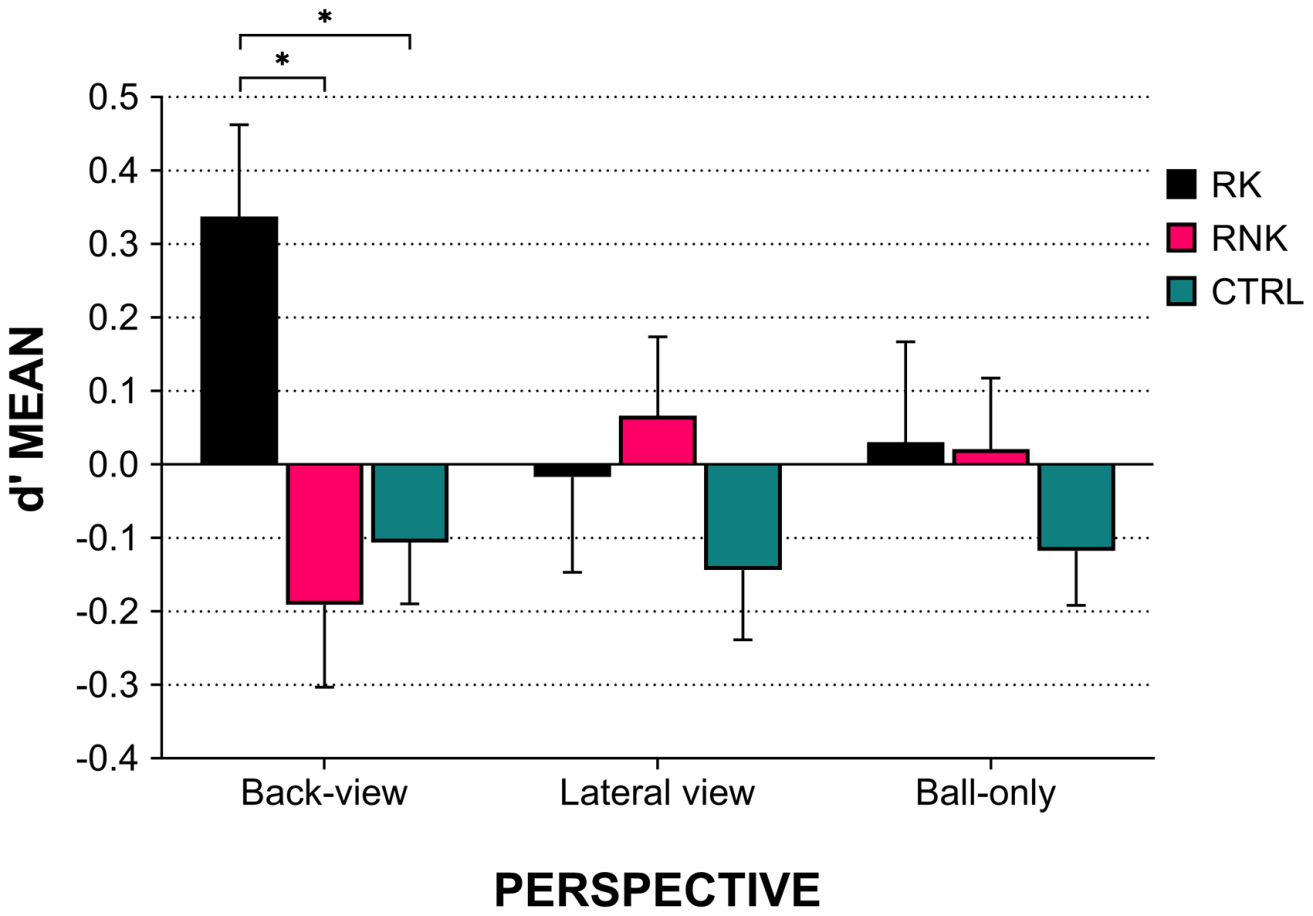
Kick outcome prediction according to group and perspective. The histograms indicate the average *d*’ scores, error bars indicate standard error. Asterisks indicate *post hoc* values of *p* < 0.05. RK, Rugby Kickers; RNK, Rugby Non-Kickers; and CTRL, control group.

The mixed ANOVA combining Group and Perspective showed no main effect of Perspective [*F*_(2,244)_ = 0.15, *p* = 0.85]. Interestingly, the interaction between Perspective and Group approached the significance threshold [*F*_(4,244)_ = 2.08, *p* = 0.08] with an effect driven almost exclusively by the RK outperforming the other groups when stimuli were presented as back-view [RK vs. RNK: *t*_(59)_ = 3.02, *p*_(corrected)_ = 0.02, Cohen’s *d* = 0.78; RK vs. CTRL: *t*_(97)_ = 3.04, *p*_(corrected)_ = 0.02, Cohen’s *d* = 0.63]. No other modulations emerged both within and across groups (Figure 3).

**Figure 4:**
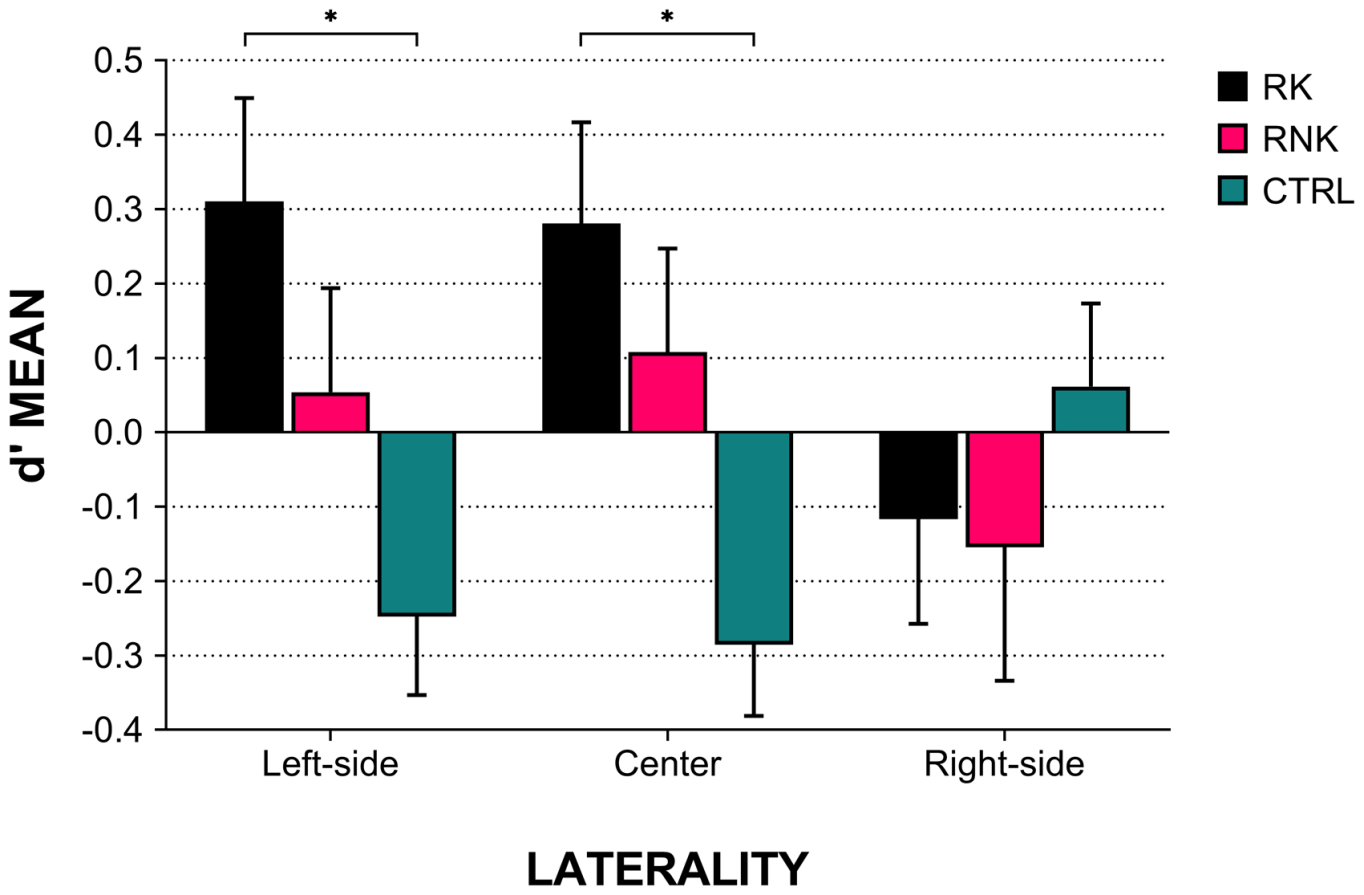
Kick outcome prediction according to group and laterality. The histograms indicate the average *d*’ scores, error bars indicate standard error. Asterisks indicate *post hoc* values of *p* < 0.05. RK, Rugby Kickers; RNK, Rugby Non-Kickers; and CTRL, control group.

No main effect was found when analyzing the Zone factor [*F*_(2,244)_ = 1.17, *p* = 0.31]. In our hypothesis, we expected a modulation driven by such a factor onto the outcome prediction, mainly reflecting a lower performance in lateral zones from which placed kicks are more challenging. However, this was not the case. Thus, we wondered whether the performance in outcome prediction could distribute along the eight positions of the court following a different organization and, namely, laterality. For a right-footed kicker, typically using the foot’s inner side to impact the ball, kicking from left-sided positions is easier than right-sided ones. Therefore, we grouped the eight positions according to their left, central, or right laterality relative to the goalposts (see dotted circles in Figure 1). While no main effect was observed in the mixed ANOVA integrating Group and Laterality [*F*_(2,244)_ = 0.63, *p* = 0.52], a significant interaction emerged [*F*_(4,244)_ = 3.70, *p* = 0.006] showing that RK was advantaged compared to CTRL in the outcome prediction when the observed kick was executed from the left-sided [*t*_(97)_ = 3.17, *p*_(corrected)_ = 0.02, Cohen’s *d* = 0.67] and central sectors [*t*_(97)_ = 3.44, *p*_(corrected)_ = 0.007, Cohen’s *d* = 0.72] of the court (see Figure 4). The results reported above were entirely confirmed after accounting for the foot-laterality of the participants.

Finally, the ANOVA considering Group and Player indicated no significant main effect of player [*F*_(3,366)_ = 0.36, *p* = 0.77] or interaction [*F*_(6,366)_ = 0.56, *p* = 0.75], suggesting a minor role of the observed model expertise in favoring the outcome prediction (see Supplementary Figure S1). However, the participant’s performance is not evenly distributed across the four players, with RK exhibiting higher performance when observing P2 than other players.

In summary, the results highlighted a better performance of the RK, especially when the stimulus is observed in the back-view and the kick is attempted from more favorable positions on the court. These aspects point to the role of both visual experience and motor expertise in promoting kick outcome prediction. To strengthen this hypothesis, we thus applied a multiple regression model to test the possible predictive role of the reported motor expertise and visual experience on the outcome prediction. Because of the lack of motor expertise inherent in the CTRL group, such analysis was limited to the pool of rugby players (RK and RNK).

Nine RK participants were excluded as they had not provided quantitative information about their motor expertise or visual experience (e.g., they declared to have served as kickers but did not indicate the range of placed kicks executed, thus impeding to rank their motor expertise). The final sample comprised 26 RK and 26 RNK (*n* = 52). Furthermore, starting from the results reported above, we limited the regression analysis to the stimuli seen from the back-view, i.e., those for which RK had outperformed other participants.

The multiple regression model combining motor expertise and visual experience showed a trend toward significance in explaining the performance in outcome prediction (*r* = 0.32, *p* = 0.06). Analyzing the individual parameters estimates, it emerged that motor expertise is a significant predictor of our participants’ performance (β = 0.30, *p* = 0.03), while the visual experience appears unrelated to the same scores (β = 0.05, *p* = 0.70).

## 4. Discussion

Starting from previous observations indicating that motor expertise set better premises for outcome predictions in sports (Rizzolatti et al., 2021), in the present study, we wondered whether the same effect could also be identified within a given sport, i.e., among teammates covering different positions. We selected the sport of rugby and placed kicks as the specific gesture because they are performed exclusively by 1–2 players in a team composed of 22 players. We recruited three groups of subjects (rugby kickers, rugby non-kickers, and non-rugby players), asking them to report their visual experience with rugby (range of watched matches) and, in the case of rugby players, their motor expertise (range of executed placed kicks). These choices allowed us to test whether placed kick outcome prediction is modulated across groups and whether modulations rely more on the visual experience or motor expertise.

Rugby kickers outperformed the other groups in terms of overall accuracy. While the comparison between kickers and controls may seem trivial and is fully in line with previous literature (Calvo-Merino et al., 2006; Aglioti et al., 2008; Urgesi et al., 2012; Gabbett and Abernethy, 2013; Makris and Urgesi, 2015; Loffing and Cañal-Bruland, 2017), the notion that rugby kickers better predict kicks outcomes compared to their non-kicking teammates reveals the fundamental role that the individual motor expertise exerts onto the capacity to decode other people behavior. Indeed, participants of both groups are well balanced in their confidence with rugby (they played several matches and attended competitions, including placed kicks), with the only difference being their direct experience with placed kicks. This seems in contrast to what emerged in a study by Runswick et al. (2020), in which the authors suggested that the players’ accuracy in predicting ball-bounce outcomes after grubber or chip kicks in rugby depends on players’ non-position-specific expertise. Thus, when bridging motor expertise with the capacity to decode others’ actions, one should not refer to all the motor engrams belonging to a given field (sport in our case) but rather to the sole ones represented in the individual motor repertoire.

In our experimental design, we introduced several factors potentially impacting the outcome prediction: Perspective, Zone/Laterality, and Player. One could hypothesize a main effect of Perspective, given the different levels of information conveyed to participants. Back-view is the richest, as movement preparation, foot-ball contact, and goalposts are fully visible. On the contrary, the Ball-only perspective is the least informative, as only the foot-ball contact is visible. Finally, the Lateral view is set at an intermediate level.

Contrary to our expectations, no main effect of Perspective emerged. However, the significant Group*Perspective interaction revealed a perspective-specific modulation among groups, with rugby kickers being more accurate than other groups limited to the back-view perspective. Here, we wondered why such modulation does not appear for lateral views, in which motor cues are similarly represented. We found an explanation in the spatial configuration of the placed kick, whose preparation and run-up develop mostly axially relative to the goalposts. This means that the back-view perspective is better at depicting these visuospatial aspects, which, however, require solid motor expertise to enhance the outcome prediction. Another possibility is that the back-view view could be easier to embody by rugby kickers, as it depicts a scenario highly resembling the one seen during the actual kicking action. In this sense, one could assimilate such a perspective to an “egocentric” one, which enhances the observer’s motor resonance (Angelini et al., 2018).

The findings reported above suggest that motor expertise might prevail over the visual experience in supporting others’ actions decoding. Another proof of this statement would be whether our participants, especially kickers, show an outcome prediction depending upon the execution difficulty of the observed action. To test this hypothesis, we presented placed kicks performed from eight different court positions, further grouped into three laterality sectors. Contrary to other groups, kickers performed better when observing kicks from the left and central sectors than right ones. Notably, this distribution parallels the execution difficulty of placed kicks. For a right-footed kicker using the foot’s inner side to impact the ball, kicking from left-sided positions is easier than right-sided ones due to the curling effect. As the same pattern was not found for the other two groups, we can advance that the modulations induced by the motor difficulty act only for experienced subjects. As a side note, opting for *d*’, we adjusted for any response bias intrinsic to the diverse zones/lateralities, ensuring that results reflected only the specific accuracy level.

Another factor potentially impacting the outcome prediction was the observed kicker and his level of expertise. No significant effect was found, suggesting a minor role of this aspect in explaining our participants’ performance. As a speculation, it is interesting to note that kickers performed better when observing senior (P1 and P2) compared to junior players (P3 and P4), with the highest performance found for P2 kicks. One could imagine that the greater efficacy of P1 and P2 videos is associated with our participants being adults, thus having a motor schema more similar to senior than junior players. Even within senior stimuli, kickers showed a different preference for P2 videos. An explanation could be that P1 is a top professional player with extremely consolidated and perfectioned kinematics, possibly making our participants more distant from his model than P2.

In summary, most of our findings point to the supremacy of motor expertise over visual experience in promoting the outcome prediction of a given sport gesture. However, this conclusion derives mainly from the comparisons between two groups of players, the former with high motor expertise and visual experience and the latter with only visual experience. Thus, the remaining points are (1) whether at the group level it is possible to highlight a causal role of motor expertise on the outcome prediction and (2) whether such dependency also resists when both visual experience and motor expertise are modeled. Our regression results strongly indicate the validity of both these assumptions. Indeed, our participants’ motor expertise significantly and positively predicted their performance, while the same was not true for the visual experience scores.

Our findings collectively indicate that our motor expertise is the fundamental substrate underlying our capacity to decode others’ behaviors and predict their outcomes. This aligns with previous studies by Calvo-Merino et al. (2006), showing that dancers activate their mirror mechanism according to their motor (but not visual) experience, and with that by Aglioti et al. (2008), proving that basketball players better predict free-throws outcome compared to journalists and controls. We extended this general principle to the case of team sports, which is highly relevant for two reasons: first, because of the possibility of selecting expert and non-expert subjects within the same team, i.e., balancing confounding factors like motivation, engagement, etc.; second, more importantly, because it is in team sports that predicting opponents and teammates behaviors plays the major role in determining the results of competitions.

Starting from these premises, we would provocatively advance a new training paradigm for team sports, in which all players should not only practice their position-specific motor gestures but also directly experience the motor gestures they are expected to interact with most frequently. Such a schema is intended to equip them with motor knowledge not replaceable by any visual information (see also Lucia et al., 2022, 2023). From this perspective, it is worth mentioning that action observation can be used to integrate motor training procedures in the acquisition of new motor skills (Rizzolatti et al., 2021; Nuara et al., 2023), especially alternating sessions of motor practice with the observation of the same gesture (Bazzini et al., 2022) and to make the visual stimuli challenging yet achievable according to the individual participant (Nuara et al., 2019; De Marco et al., 2020).

A few limitations must be disclosed about our study, and they mainly pertain to the specificity and generalizability of the findings. As far as the first point is concerned, the experimental procedures did not include a non-rugby-related action prediction (e.g., grasping with different intentions, De Marco et al., 2020) or general cognitive task (e.g., Stroop Test), thus impeding to evaluate quantitatively whether the better performance of kickers is specific for their experienced gesture or rather extends to multiple visuomotor or even cognitive domains. This latter point would postulate that the sampled groups are unbalanced in terms of cognitive profiles, for instance, in attentive or visuospatial skills. Although we cannot rule out this point, the sample size of our groups makes this hypothesis quite unplausible, and the training experience highly shared between kickers and non-kickers further temper this possible criticism. On top of this issue, one could wonder whether our findings are indeed generalizable to any motor domain, including non-sport-related ones. Caution is mandatory, yet multiple clues support this view. Sports is the ideal context for testing the highest segregation of motor repertoires, as naïve people often never attempted the targeted gestures. This is not true for everyday gestures like manipulative actions, as some degree of visuomotor exposition stands for everybody. Alongside, neurophysiological evidence indicated that the acquisition of basic motor competencies like crawling or walking determines a higher or lower motor resonance during the observation of that action (van Elk et al., 2008). Considering all these factors, we are confident that a similar principle applies to motor skills in general, even if further studies will have to tackle this aspect longitudinally to multiple motor competencies.

## Data availability statement

The raw data supporting the conclusions of this article will be made available by the authors, without undue reservation.

## Ethics statement

The studies involving human participants were reviewed and approved by Commissione per l’Etica e l’Integrità nella Ricerca—National Research Council (CNR), n. 00111709/2022, 15.02.2022. The patients/participants provided their written informed consent to participate in this study. Written informed consent was obtained from the individual(s) for the publication of any potentially identifiable images or data included in this article.

## Author contributions

SP designed the study, performed the research, analyzed data, and wrote and revised the manuscript. MCB analyzed data and wrote and revised the manuscript. MR conducted the research. DDM, AN, PP, and ES revised the manuscript. PA and MF-D designed the study and wrote and revised the manuscript. All authors contributed to the article and approved the submitted version.

## Funding

The study has been partially supported by a grant from Fondazione Cariparma to IN-CNR (ROL 2021/12296).

## Conflict of interest

The authors declare that the research was conducted in the absence of any commercial or financial relationships that could be construed as a potential conflict of interest.

## Publisher’s note

All claims expressed in this article are solely those of the authors and do not necessarily represent those of their affiliated organizations, or those of the publisher, the editors and the reviewers. Any product that may be evaluated in this article, or claim that may be made by its manufacturer, is not guaranteed or endorsed by the publisher.

## Footnotes

^1^ Prolific, https://prolific.co/

^2^ Gorilla Experiment Builder, http://www.gorilla.sc/
