# Supplementary material for: Kicking in or kicking out? The role of the individual motor expertise in predicting the outcome of rugby actions
Source: Front Psychol. 2023 Mar 3;14:1122236. doi: 10.3389/fpsyg.2023.1122236 (PMC10020490; doi:10.3389/fpsyg.2023.1122236)
Supplement: Supplementary file 1 [file Table_1.DOCX]

SUPPLEMENTARY MATERIALS


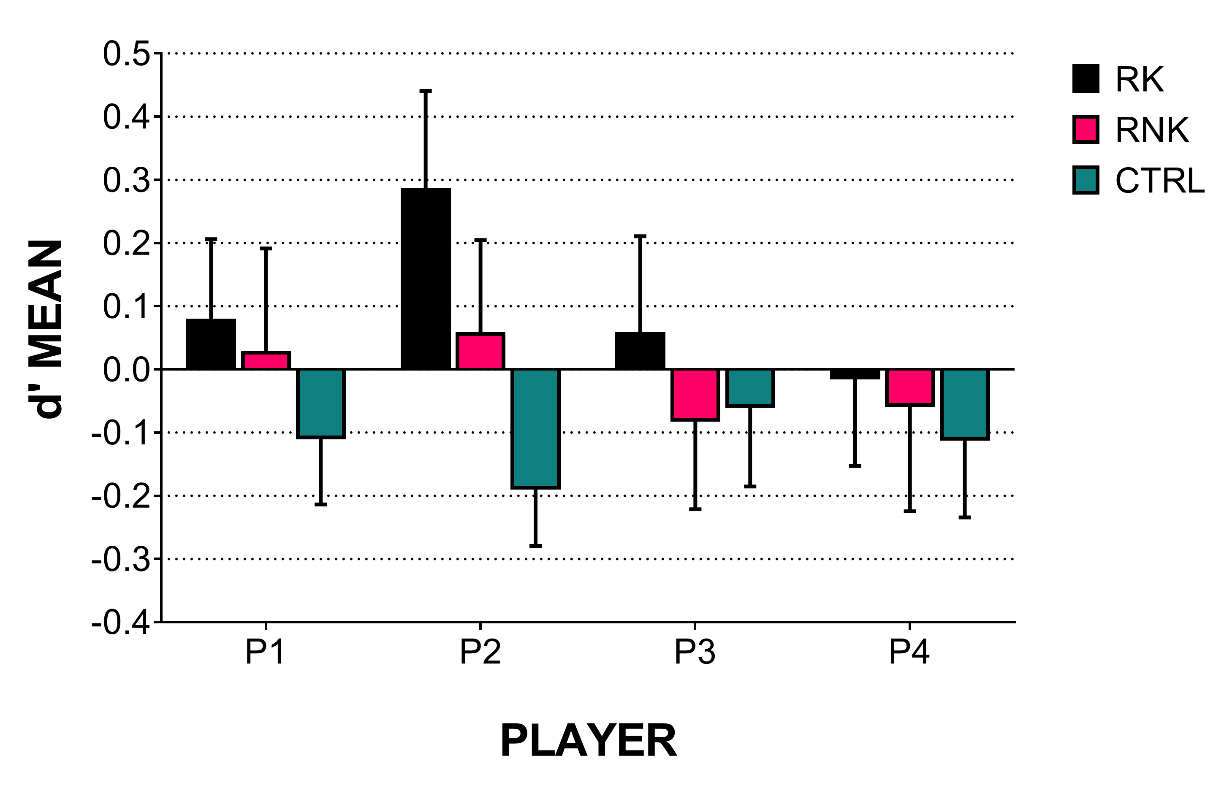


Figure S1: Kick outcome prediction according to group and player. The histograms indicate the average d’ scores, error bars indicate standard error. RK: Rugby Kickers; RNK: Rugby Non-Kickers; P: player; CTRL: control group.
